# Network and matrix analysis of the respiratory disease interactome

**DOI:** 10.1186/1752-0509-8-34

**Published:** 2014-03-22

**Authors:** Benjamin Garcia, Gargi Datta, Gregory P Cosgrove, Michael Strong

**Affiliations:** 1 Integrated Center for Genes, Environment, and Health, National Jewish Health, Denver, CO 80206, USA; 2 Computational Bioscience Program, University of Colorado Denver, Anschutz Medical Campus, Aurora, CO 80045, USA; 3 NICTA, Victoria Research Lab, Melbourne, Victoria 3010, Australia; 4 Department of Medicine, National Jewish Health, Denver, CO 80206, USA

**Keywords:** Interactome, Networks, Respiratory diseases, Lung disease

## Abstract

**Background:**

Although respiratory diseases exhibit in a wide array of clinical manifestations, certain respiratory diseases may share related genetic mechanisms or may be influenced by similar chemical stimuli. Here we explore and infer relationships among genes, diseases, and chemicals using network and matrix based clustering methods.

**Results:**

In order to better understand and elucidate these shared genetic mechanisms and chemical relationships we analyzed a comprehensive collection of gene, disease, and chemical relationships pertinent to respiratory disease, using network and matrix based analysis approaches. Our methods enabled us to analyze relationships and make biological inferences among over 200 different respiratory and related diseases, involving thousands of gene-chemical-disease relationships.

**Conclusions:**

The resulting networks provided insight into shared mechanisms of respiratory disease and in some cases suggest novel targets or repurposed drug strategies.

## Background

The capability to catalog interactions among diseases, chemicals, and genes into well-curated databases offers a collective knowledge of experimental results that has great potential for the generation of hypotheses and meta-analyses. To date, many biological databases have been established to catalog relationships among genes [1], diseases [2], and chemicals [3]. Many of these databases focus on one particular type of relational interaction, ranging from protein-protein interaction databases [1], gene-chemical databases [4], and disease-gene databases [2], and are often constructed using data mining methods complemented by manual curation. The described databases, in many instances, serve as the foundation for a wide array of predictive and analytical methods to examine interactions. They can also be extended to analyze interactions among overarching themes, including analyzing gene-chemical interactions within the context of a given set of diseases or protein-protein interactions within the context of peptide recognition [5,6]. Integration of multiple sources and types of relational data remains an important and challenging research area with great potential toward the development of furthering our understanding complex diseases and interactions.

Each year over 400,000 deaths occur in the United States as a result of respiratory and related diseases (RRD) [7]. Given the high prevalence and importance of lung and respiratory diseases, we hypothesized that a better understanding of the respiratory gene-chemical-disease interactome would lead to better understanding of the molecular mechanisms of lung disease, including the environmental and drug influences, and more importantly, may lead to new treatment or intervention strategies. In this study, we focus our efforts on the analysis of gene-disease-chemical relationships, in order to elucidate and infer novel interactions and to understand biology pertinent to respiratory diseases using network and matrix-based methods.

Current network and matrix-based analyses of disease relationships has relied heavily on gene or protein-centric examinations [8-11], neglecting chemical features that may also influence disease. Likewise, network analysis techniques have often been developed and utilized to examine gene or protein relationships among diseases [12], but often neglect environmental or chemical factors that may influence disease. In cases where genes, diseases, and chemicals have been analyzed, often the networks are decoupled to allow for the analysis of a single entity or relationship type, such as the effect of a drug on a gene network or the elucidation of molecular mechanisms in disease [13-15]. Host-pathogen studies have also largely focused on a single relational type, predominantly protein-protein interaction relationships [16]. Here we apply methods to investigate gene-chemical-disease networks, in order to better understand the genetic and chemical contributors of diseases, elucidating novel biology and helping to further understand shared disease pathology.

## Results and discussion

### Network construction

In order to compile a comprehensive dataset to examine gene, disease, and chemical relationships pertinent to respiratory disease, we extracted information from the Comparative Toxicogenomics Database (CTD) [4] and the Human Protein Reference Database (HPRD). CTD houses manually curated information pertinent to gene-disease-chemical relationships for a wide variety of diseases, and HPRD houses information focusing on protein-protein interactions from a wide array of experiments in humans and other model organisms. CTD offers a conservative and expert curated source of interactions to form networks, and HPRD uses the same normalized gene names as CTD.

We compiled and filtered our in-house database in two ways. The first database, we refer to as the whole respiratory network (Additional file 1: Table S1), and the second database we refer to as the therapeutic network (Additional file 2: Table S2). The whole respiratory network represents disease-gene, disease-chemical, chemical-gene, and gene-gene interactions associated with respiratory diseases. The therapeutic network, in contrast, consists of a subset of the respiratory network, containing only chemicals with curated therapeutic interactions with diseases and the genes that interact with those chemicals. These curated therapeutic interactions are established using the “DirectEvidence” field from CTD. This network was called the therapeutic network as a reference to this inclusion criterion. In addition to the therapeutic inclusion criteria, chemical-chemical interactions were also included based upon curated chemical relationships derived from chemical gene-interaction information. Gene-gene interactions were established using the HPRD database [1].

To assess the directionality of chemical-gene interactions, the uniqueness of chemical-gene and gene-chemical interactions were assessed. First, chemicals with disease interactions were batched queried using CTD, with an output of curated chemical-gene interactions. Second, genes with disease interactions were batched queried using CTD, with an output of curated gene-chemical interactions. The intersection between these two sets was then calculated. In the whole respiratory network, there were 27075 total chemical-gene and gene-chemical linkages with 13543 remaining after accounting for bi-directionality of interactions. Given the small percentage of directional linkages (~0.05%), all links were treated as bi-directional.

The type of interaction was established for disease-chemical, disease-gene, and chemical-gene interactions. For disease-chemical and disease-gene interactions, there were three types of interactions based upon CTD curation: therapeutic, marker/mechanism, and both therapeutic and marker/mechanism. Chemical-gene interactions had three major effects and one minor effect based upon CTD curation. The major effects are increasing, decreasing, and affecting expression or activity. The minor effect is based upon the type of protein modification imparted by the chemical onto the protein. The list of protein modification includes: ubiquitination, phosphorylation, oxidation, cleavage, methylation, hydrolysis, hydroxylation, glycosylation, glucuronidation, acetylation, nitrosation, ribosylation.

To establish chemical-chemical linkages and the type of gene-chemical linkage, CTD was used [4]. Chemical-gene interactions were extracted with a query specifying interaction type. Co-interactions between multiple chemicals and a gene were extracted from this list and chemical-chemical linkages were established if two chemicals had a curated co-occurrence with a gene. A co-occurrence was determined when a secondary chemical appeared in the interaction characteristics between a chemical and a gene. The type of linkage between the two chemicals was classified using the same type of link used to classify chemical-gene interaction in which the co-occurrence appeared. As there is often discordance between the naming of chemicals, especially those with pharmaceutical implications, a chemical reaction database and drug interaction database were not utilized for establishing chemical-chemical interactions.

**Figure 1:**
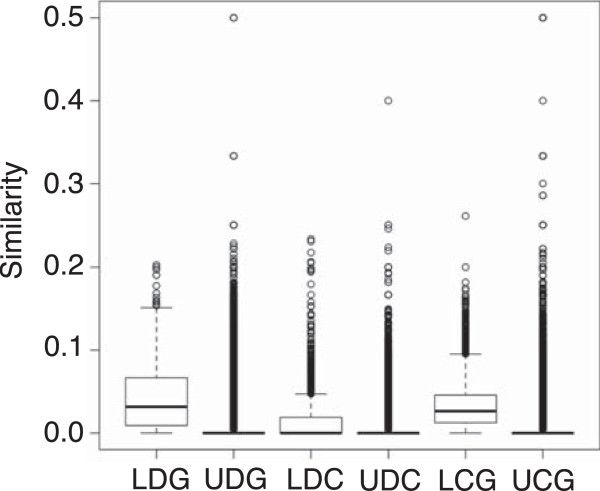
**Similarity of linked and unlinked nodes.** Jaccard similarity coefficients between linked and unlinked nodes in the therapeutic network. LDG – linked disease-gene, UDG – unlinked disease-gene, LDC – linked disease-chemical, UDC – unlinked disease-chemical, LCG – linked chemical-gene, UCG – unlinked chemical-gene. In all three sets, the linked nodes were more similar than unlinked nodes determined by Mann Whitney U tests (p < 0.01). The median for all unlinked node pairs is 0, with the medians for the linked pairs: DG – 0.026, DC – 0, CG – 0.032.

After construction of the network, Jaccard similarity coefficients were generated between all nodes. Each coefficient was then classified based upon whether the two nodes were connected and the type of nodes being connected. Figure 1 represents the three node interaction types of interest: disease-gene interactions, disease-chemical interactions, and chemical-gene interactions. To test the alternative hypothesis that linked nodes are more similar than unlinked nodes based upon a Jaccard coefficient, Mann–Whitney U tests were performed on each of the three sets with a null hypothesis that the similarity between linked nodes and unlinked nodes is the same. In all three cases, Mann–Whitney U tests showed with greater than 99.9% confidence that linked nodes were more similar than unlinked nodes (p < 0.01). This suggests that the greater the similarity between nodes, the more likely they are to interact. To assess the stability of the Jaccard coefficient, single edge additions were added to sub-networks. Kolmogorov-Smirnov tests were then run on the Jaccard coefficient distributions of the individual sub-network against perturbations within that sub-network. The result is that no perturbation caused a significant shift in distribution (average p-value ~ 0.99), with smaller sub-networks being more affected by perturbations (minimum p-value ~ 0.10). This lack of significant change is due to an addition of one edge having only small impacts on network topology, validating the Jaccard similarity as a stable measure of similarity for small amounts of missing data.

### Clustering methods

Evaluation of protein-protein interaction network clustering methods is generally performed through the comparison of gold standard regulatory networks or pathways. Since an analogous gold standard gene-chemical-disease network does not exist, for us to evaluate clustering methods, we selected high performing methods used for clustering protein-protein interaction networks, with the added stipulation that their output is scalable to a more sparse and dissimilar network. MCODE and MCL, two widely accepted and utilized clustering methods were tested for scalability when adding additional node types [11,17-19]. The gene-gene portion of the network was used as a baseline for the types and sizes of sub-networks that can be expected in an ideal situation. In the gene-gene network, both algorithms performed similarly with median sub-network sizes of 4 for MCODE and 3 for MCL. In the larger sub-networks both methods displayed highly interconnected clusters. In the therapeutic network, however, the clustering methods performed much differently. MCODE had a median sub-network size of 18, while still maintaining the highly interconnected networks, and MCL had a median cluster size of 3, and no longer exhibited an interconnected feature. We also applied hierarchical clustering, utilizing a Pearson’s correlation coefficient. Pearson’s has been shown to be a highly robust unsupervised correlation that performs well under a multitude of protein-protein interaction analyses, from identifying regulatory networks to identifying groups of proteins with shared functions [20,21]. A lack of a gold standard gene-disease-chemical network is also why no semi-supervised or supervised methods were chosen.

### Node-edge analysis

For the whole respiratory network, nodes were input based upon type (disease, chemical, gene) and edges based upon types of nodes involved (disease-gene, gene-gene, chemical-gene, disease-chemical) into Cytoscape [22], creating a network of 1,830 nodes and 17,275 edges. This network became a test-bed for methods to improve subsequent analyses including constructing networks with only one type of edge, and networks with filtered diseases, chemicals, and genes of interest. These tests led to the creation of both a gene-disease sub-network and the therapeutic chemical network.

**Figure 2:**
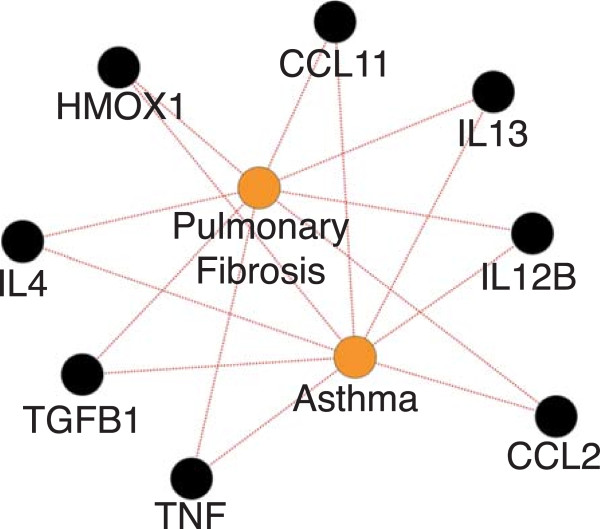
**Gene-disease sub-network.** Shared genes between Pulmonary Fibrosis and Asthma. Many of these genes represent similar pathophysiology in both diseases, such as activation of Th2 cell inflammation by IL13 and IL4. Red links represent marker/mechanism links.

The gene-disease sub-network was visualized by Cytoscape to determine clusters of similar genes not seen in the overall network. Figure 2 shows one such cluster of shared genes between asthma and pulmonary fibrosis. Among the genes we observe linked to pulmonary fibrosis and asthma, we see the IL4 and IL13 cytokines. Both IL4 and IL13 are involved in activating Th2 cell inflammation, involved in asthma. Both IL4 and IL13 antagonists have also been shown to be effective in asthma therapy through the dampening of inflammation associated with asthma. In addition to being involved in asthma, IL13 has also been linked to pulmonary fibrosis, stimulated by the activation of Th2 cell inflammation, leading to tissue fibrosis. TGFB1 also induces inflammation, apoptosis, and fibrosis in mouse models [23,24], and has been associated with asthma. Networks such as these may be used to identify shared genetic mechanisms or molecular pathways of disease, and can also be used to identify novel drug targets or repurposed drug strategies to combat diseases that may be clinically very different, but that may share common genetic or molecular relationships.

**Figure 3:**
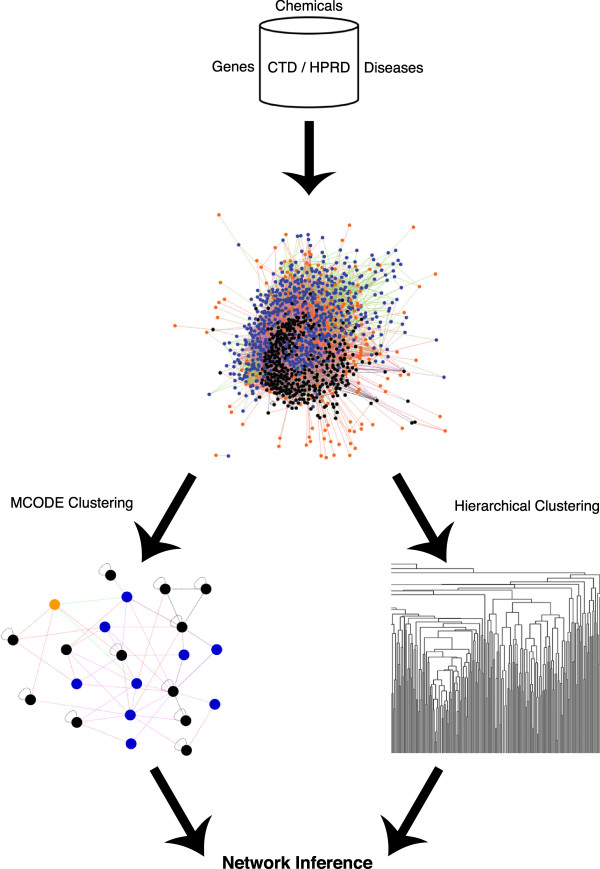
**Network analysis flowchart.** Interaction development pipeline for extracting interaction and node information from CTD and HPRD, construction of network from interaction information, creating sub-networks from the network and clusters from the matrix, and identifying biologically relevant information to make novel inferences.

For the therapeutic network, full information about the interaction between nodes was input into Cytoscape and visualized using an organic graph layout [22]. Nodes were colored by disease, chemical, or gene. Edges were colored by positive interactions (therapeutic or increases), negative interactions (marker/mechanism or decreases), mixed interactions (affects or therapeutic with marker/mechanism), and color intensity weighted by any protein modifications. Based upon database inclusion criteria, there were 388 genes, 227 diseases, and 578 chemicals. There were 10,679 linkages between these nodes, with each linkage having a characteristic path length of 3 and each node having an average of 18 neighbors. These numbers are about half that of the whole respiratory network, both decreasing the size of the network and making the network more directed towards finding positive interactions between chemicals and diseases. Linkage statistics from both networks can be seen in Table 1. A schematic of the overall process of creating and analyzing the therapeutic network can be seen in Figure 3.

**Table 1:** Network nodes and links

	**Whole respiratory**	**Therapeutic**
Nodes		
Genes	426	388
Chemicals	1177	578
Diseases	227	227
Total	1830	1193
		
Links		
Gene-chemical	13543	7587
Gene-gene	438	433
Chemical-chemical	0	435
Disease-gene	577	536
Disease-chemical	2717	1688
Total	17275	10679

Counts of each type of node and linkage for both the whole respiratory network and the therapeutic network.

**Figure 4:**
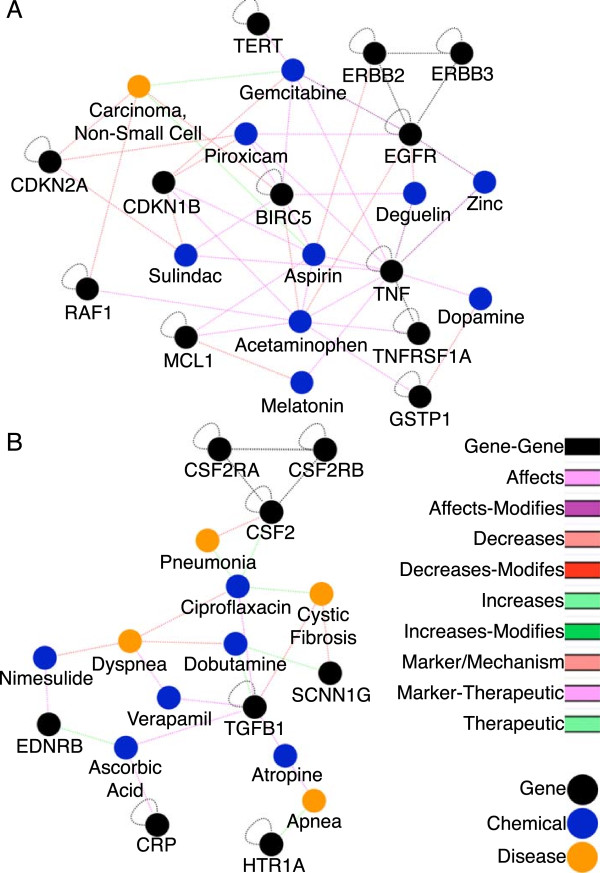
**Visualization of sub-network interactions. A)** Clustered sub-network containing non-small-cell lung carcinoma and a restricted view of closely interacting chemicals and genes for visualization purposes. **B)** Clustered sub-network containing Cystic Fibrosis and closely interacting diseases, chemicals, and genes.

To elucidate clusters of interest, the Cytoscape plugin MCODE was run on the network using a degree cutoff of 2, a node score cutoff of 0.2, a K-Core of 2, and a max depth of 100 [17]. This resulted in 18 highly interconnected clusters with a diverse set of node types (Additional file 3: Table S3), allowing the therapeutic network to be investigated and parsed into manageable sub-networks. These sub-networks offer a more manageable network to elucidate and identify novel and relevant interactions. Figure 4 demonstrates two of these sub-networks. Non-connected nodes that occur in highly interconnected sub-networks, particularly those with shared neighbors, offer a refined starting point for inferring novel interactions. Connections of interest were investigated by randomly choosing 23 unlinked node-pairs from the resulting sub-networks. These 23 inferred links were then analyzed by manually mining literature and databases for evidence that the two nodes might be linked by methods beyond those we used to establish our networks. In the absence of a gold standard, manual literature mining is often used to for validating inferences [25]. Supporting evidence for these inferred links can be seen in Table 2.

**Table 2:** Inferred interaction summary

**Link**	**Inferred links**	**Literature support**	**Expression support**	**Database support**	**Anti-support**	**No support**
Gene-disease	10	9	0	3	0	1
Gene-chemical	9	3	5	1	1	4
Chemical-disease	4	2	0	1	1	0

Type of inferred link and support for each link. Inferred link is number of currently non-linked node pairs analyzed in each category. Literature support means a PubMed search resulted in a published article that supports the link. Expression support means there is literature support for gene expression changes. Database support means that there is support for a link due to curation methodology or the link was added in later version of CTD. Anti-support means that literature specifically says this link is not real, and no support means that no evidence could be found for or against the link.

One of these sub-networks, shown in Figure 4A, contains non-small-cell lung carcinoma and closely interacting genes and chemicals. From this sub-network, three links were analyzed in greater detail: aspirin - EGFR, acetaminophen - non-small-cell carcinoma, and piroxicam - non-small-cell carcinoma. Aspirin - EGFR is an inferred link in this sub-network that was added as a direct link to an update of CTD that occurred after the creation of this network [4]. There was strong support in literature for aspirin promoting EGFR inhibitors, enough for a curated interaction between these two elements [26,27]. This link represents a verified prediction both by literature and by CTD, representing the effectiveness of using sub-networks to find novel links. Acetaminophen - Non-small-cell lung carcinoma is a link that has negative support in literature [28]. In studies involving testing multiple anti-inflammatory drugs for change in non-small-cell lung carcinoma outcome, they found no correlation between Acetaminophen and change in prognosis [28]. The negative support for this link shows that while sub-networks offer a starting point for testing inferred interactions, not all of the nodes will have a direct link. Lastly, Piroxicam - non-small-cell carcinoma had direct and indirect literature support for this link [29,30]. There was increased immune function in lung cancer patients that had piroxicam added to their drug regimens [29]. Also, piroxicam showed decreased tumorigenesis in mice with colon cancer, suggesting this link might be present in other cancers as well [30]. This link represents a possible avenue for further research. There is evidence to support that there are beneficial effects of piroxicam on non-small-cell carcinoma prognosis; however, the full effects of this interaction are not well understood.

Analysis of sub-networks also presents the ability to find links for similar or comorbid diseases. In the cystic fibrosis sub-network, Figure 4B, dobutamine interacts with both of cystic fibrosis’ genes in the sub-network, suggesting a link between dobutamine and cystic fibrosis. Upon searching the literature, dobutamine, especially in combination with nitric oxide, improves pulmonary hypertension in cystic fibrosis patients, a common comorbidity [31]. CTD neither contains a link between dobutamine and cystic fibrosis nor dobutamine and pulmonary hypertension.

Jaccard similarity coefficients were generated for each sub-network. These coefficients measured similarity using only nodes and links present within the sub-network. Similarities were then averaged for each node, representing how similar a given node is to the sub-network as a whole. The same 23 unlinked node pairs from the previous analysis were used to determine the relationship between similarity and literature evidence. Similarity between the two nodes was ranked against the similarity of all other pairwise Jaccard coefficients within the sub-network, with the similarity being broken into one of three sets: upper 25^th^ percentile, middle percentile, and the lower 25^th^ percentile. These comparisons represent how similar the two nodes are to each other, relative to the sub-network as a whole. Evidence for a possible interaction was then manually mined from published articles, and then compared to their similarity classifications. Table 3 represents mined literature support against similarity classification. With increasing similarity between the two nodes, relative to their ranked similarities within the sub-network, there was increasing evidence in literature to support connection between the two nodes. In addition to having a greater likelihood of evidence based upon similarity, just being in the same sub-network increased the likelihood of two nodes having a connection over the 0.015 probability of any two random nodes being linked together in the databases used for constructing the network. This shows a complimentary relationship between clustering and similarity when trying to determine if there is evidence to support two nodes being linked.

**Table 3:** Jaccard similarity assessment

**Jaccard percentile**	**Support**	**Anti-support**	**No support**	**Percent support**
75-100	7	1	1	77.8%
25-75	5	0	2	71.4%
0-25	4	1	2	57.1%

Supporting evidence for and inferred linkage utilizing the Jaccard coefficient between two nodes. The rank of the pairwise Jaccard coefficient within the sub-network was compared to the ability to find evidence supporting the pairwise connection. A rank of 100 represents the highest Jaccard coefficient within the sub-network and a rank of 0 represents the lowest Jaccard coefficient within the sub-network.

A more systematic evaluation of the relationship between Jaccard similarity and identifying novel links was performed on a human signaling network [32]. Protein-Protein interactions from the human signaling network were selected based upon both the interacting genes being present in the therapeutic network while their interaction was not present in the network. While self-interacting genes were utilized in generating Jaccard similarity values, they were excluded from both the background and the human signaling network during the analysis. This is due to the fact there is no way to distinguish between likely self-interactions and unlikely self-interactions using a similarity measure that will always be 1.0 in the case of a self-interaction. This selection resulted in 1057 additional interactions for use in validation.

**Figure 5:**
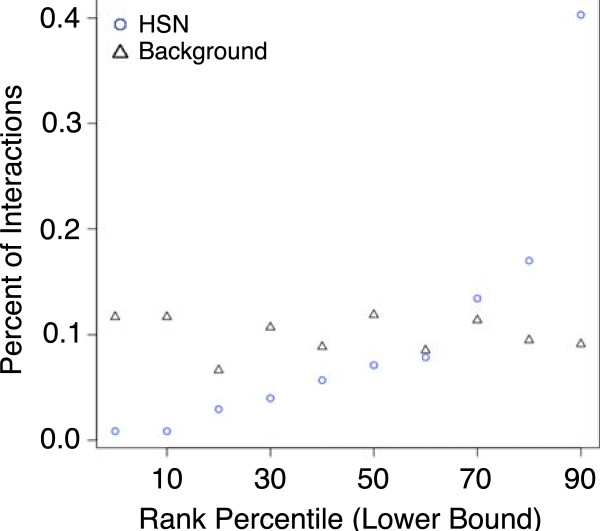
**Jaccard rank of human signaling network interactions.** Percent of gene-gene interactions that fall into a given percentile range (broken into ranges of 10 percent) for both the background of possible interactions and the novel interactions represented by the human signaling network (HSN). A lower bound of the 10^th^ percentile represents the range of greater than the 10^th^ percentile to less than or equal to the 20^th^ percentile. The discrepancy in that the background percentiles are not exactly equal to 10% is due to the fact that duplicates of Jaccard coefficients at the boundary percentiles were treated the same as the boundary.

A Mann–Whitney U test was performed on the human signaling network gene-gene interactions with the null hypothesis that there is no similarity difference from the background of possible gene-gene interactions. The alternative hypothesis is that the novel interactions from the human signaling interactions are more similar than the background. This test resulted in a p < 0.01, showing that these novel interactions are more similar than the background. Just as the literature study, rank of the Jaccard coefficient was also important to whether or not an interaction was found. There was an exponential relationship between the rank and inclusion into the human signaling network with roughly 40% of the additional interactions being in the 90^th^ percentile or greater (Figure 5).

### Matrix analysis

**Figure 6:**
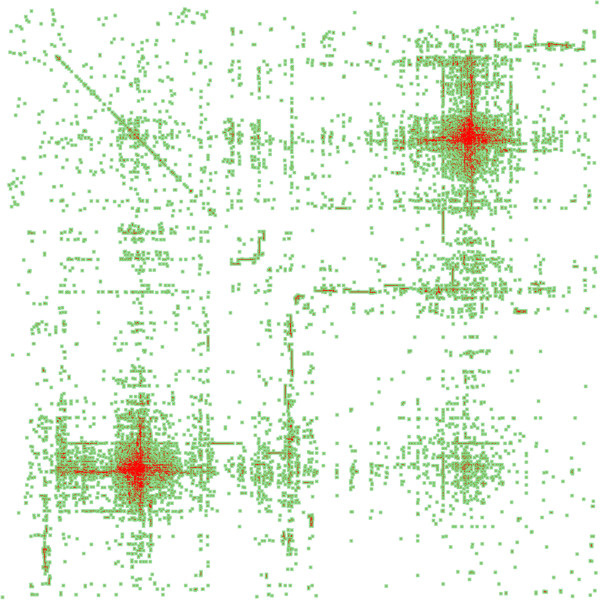
**Therapeutic diffusion matrix.** Therapeutic matrix clustered based upon uncentered Pearson’s correlation coefficient with average linkages and then hierarchical clustering. Each red dot represents an interaction pair with the rows and columns representing nodes. The green represents diffusion to aid in visualizing the sparse network. Node ordering is based upon similarity to adjacent nodes.

A binary interaction matrix was created using the network interaction triples for both the whole respiratory and therapeutic networks, Values of 1 represent an interaction; whereas, values of 0 represent a lack of interactions. These matrixes were then used as input to Cluster 3.0, an open source clustering tool [33]. An uncentered similarity matrix with average linkage was used to calculate hierarchical clustering. Output of the dendrogram was viewed in TreeView [34]. Clustered interactions from the therapeutic matrix are shown in Figure 6.

**Figure 7:**
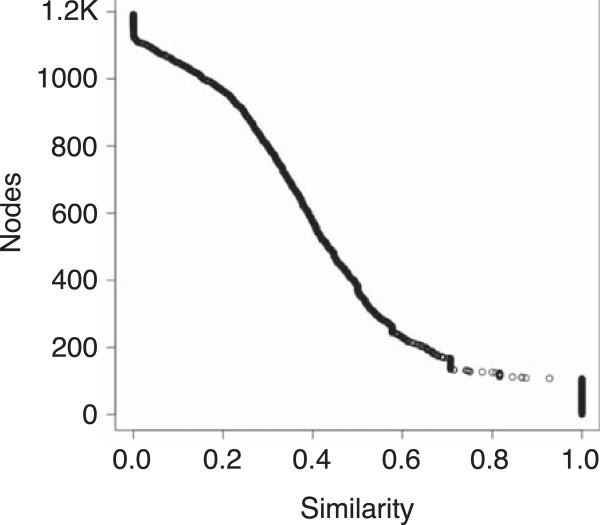
**Matrix similarity graph.** Number of hierarchical clustered nodes represented by a static cutoff for a given uncentered Pearson’s correlation coefficient. The inflection point at ~ 0.4 similarity was used to generate clusters for analysis.

Individual clusters from the therapeutic matrix were established using a 0.7 and 0.4 similarity threshold. Both of these thresholds were chosen as they represent inflection points in the node count versus similarity graph, as shown in Figure 7. Inflection points represent possible changes in cluster characteristics, such as separating high similarity clusters with medium similarity clusters. The 0.7 threshold resulted in 71 clusters. The smallest cluster had 2 nodes and the largest with 13 nodes. The 0.4 threshold resulted in 211 clusters (Additional file 4: Table S4). The smallest cluster had 2 nodes and the largest with 45 nodes. The 0.7 threshold offers the highest similarity between nodes; however, it often results in the inclusion of nodes that only have a few total number of interactions. The ERBB gene family was found in the 0.4 threshold but not in the 0.7 threshold. Also, the 0.4 threshold included both expansions and additions of clusters, such as the expansion of and anti-histamine cluster to include additional anti-histamines, and the addition of a tumorigenesis gene cluster. This expanded set of clusters supports the idea that the 0.4 threshold is more useful for finding clusters of similar function, while still maintaining a similar specificity as the clusters found in the 0.7 threshold.

**Figure 8:**
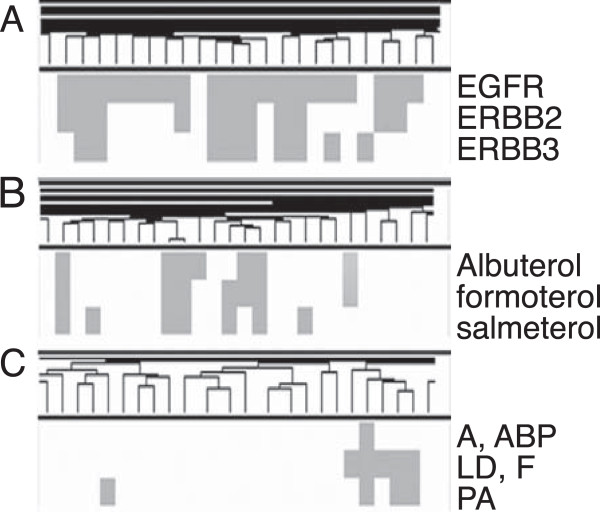
**Clusters of elements from 0.4 similarity cutoff. A)** Beta2-agonists **B)** ERBB family proteins. **C)** Fungal lung diseases; A, ABP – Aspergillosis, Allergic Bronchopulmonary; LD, F – Lung Disease, Fungal; PA – Pulmonary Aspergillosis.

Unlike sub-networks, clustering of the matrix elucidates families of chemicals, genes, and diseases with similar phenotypes and chemical characteristics. Figure 8 shows clusters in each of these three node categories from a similarity cutoff of 0.4. These clusters contain a group of beta2-agonists (Figure 8A), ERBB family proteins (Figure 8B), and a group of fungal lung diseases (Figure 8C). For the matrix clusters, genes had a tendency to cluster with other genes, chemicals with other chemicals, and diseases with other diseases. Almost all of the clusters were made up of elements of the same type, supporting the idea that this matrix clustering approach is suitable for finding nodes with similar properties versus the more diverse interactomes in the traditional sub-networks.

The assertion that subclusters can identify nodes with similar properties can be used for predicting interactions by analyzing overlap between cluster nodes and their shared interactions. In a cluster containing SEPP1, GJB1, SELENBP1, SLC22A18, A2M, and PDFGA, five out of the six genes in this cluster have an association with lung neoplasms. PDGFA, the gene not linked with lung neoplasms, has associations with breast, prostate, head and neck, and pancreas cancers. In addition, PDGFA increases with asbestos exposure, a chemical linked to mesothelioma [35]. This increase is also associated with tumorigenicity, supporting the assertion that PDFGA is also a marker for lung neoplasms [35].

Ebastine, levocabastine, hydroxyzine, SUN1334H, azelastine, olopatadine, cetirizine, desloratadine, sho-seiryu-to, epinastine, and tripolidine are a group of anti-histamine drugs that target HRH1, all of which also have interactions with rhinitis. These anti-histamine drugs also have anti-inflammatory properties, revealed by seven drugs having links to IL4, four having links to IL5, and four having links to IL8. This is supported by a study that shows various anti-histamines having anti-inflammatory properties in rhinitis pathology [36].

MT2, MT1, CCL9, CCL8, ECM1, and SLC39A4 represent a diverse cluster of two metallothionein proteins, two macrophage proteins, one extracellular matrix protein, and one zinc transporter protein. Many of these genes regulate metal concentrations within cells and are linked to respiratory hypersensitivity. Out of the five shared chemicals, only acetaminophen is linked to respiratory hypersensitivity. However, four out of these five chemicals have links to asthma, suggesting they may play a greater role in respiratory hypersensitivity in general. This hypothesis is supported by the fact that zinc deficiency alters respiratory epithelium in allergic response of mice [37].

**Figure 9:**
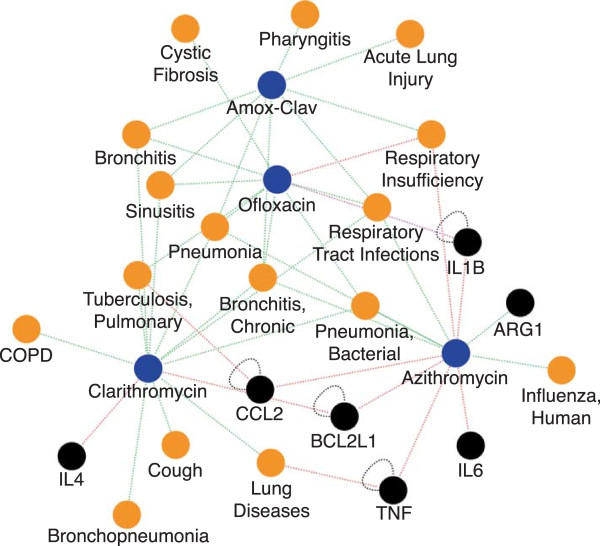
**Matrix cluster interactome.** Cluster of oflaxacin, amoxicillin-clavulanate (Amox-Clav), azithromycin, and clarithromycin with closely interacting genes and diseases.

Ofloxacin, amoxicillin clavulanate, clarithromycin, and azizthromycin are a group of antibiotics that treat respiratory infections. The interactome of these antibiotics is shown in Figure 9. Of these antibiotics, only clarithromycin and ofloxacin have direct links to tuberculosis in CTD. The drug combination amoxicillin-clavulanate has literature support that it is effective in treating tuberculosis, whereas; amoxicillin alone is ineffective [38,39]. This increase in effectiveness with clavulanate is due to the fact clavulanate inhibits an enzyme that makes *Mycobacterium tuberculosis* resistant to amoxicillin [38,39]. While literature shows that azithromycin alone is also ineffective in treating tuberculosis isolates, literature shows that azithromycin in combination with capreomycon, pyrazinamide, ethambutol, and isoniazid improves outcomes in multi-drug resistant patients over streptomycin, ethambutol, pyrazinamide, and isoniazid [40,41]. Given the fact that tuberculosis is often treated with a combination of drugs, further evaluation of amoxicillin-clavulanate and azithromycin within the context of a drug regimen would offer a more practical approach to evaluating the effectiveness of treating tuberculosis patients with these antibiotics. Also of note are the links from azithromycin and clarithromycin to IL6 and IL4 respectively. It is thought that even though azithromycin does not directly kill *M. tuberculosis* in cell culture, it may have a pro-immune effects that improves outcomes of tuberculosis patients, or may play a role as an anti-inflammatory. BCL2L1 is affected by clarithromycin, a known tuberculosis drug, and azithromycin, an inferred TB drug. This coupled with a shared interaction of CCL2 between tuberculosis and azithromycin promotes that idea that azithromycin may have a therapeutic effect on tuberculosis through an anti-inflammatory response. Through the analysis of gene-disease-chemical networks we may gain better insight into both the direct target and off target activities of certain drugs, useful in the identification of drug repurposing strategies.

### Node-edge versus matrix

While these two approaches take the same input, clustering produces two distinct results. Only eight of the eighteen sub-networks contained a cluster from the matrix where at least 50% of the nodes present in the matrix cluster were also present in the sub-network. Most of the matrix clusters that overlapped with the sub-networks contained only two or three nodes. However, one sub-network contained 11 of the 28 nodes in one matrix subcluster, making it the most nodes shared between a sub-network and a matrix cluster. These differences can be attributed to both network construction and the types of interactions that are obtained from each approach. Given the sparsity of the network, especially in chemical-chemical interactions, and the lack of disease-disease interactions, clustering coefficients and pairwise comparisons produce non-overlapping results. Clustering coefficients from node-edge based approaches represent closely interacting genes, chemicals, and diseases. These closely interacting nodes offer avenues of exploration for finding novel interactions. Pairwise comparisons from matrixes represent nodes that share the same interaction profile. This interaction profile can then be used for determining both biological meaning and novel interactions for any pairs between the cluster nodes and the interaction profile nodes. Thus, these two approaches offer a complimentary analysis strategy for sparse networks, enabling elucidation of both novel interactions and increasing our biological understanding of node clusters.

The second distinction these two approaches offer is in the visualization of interactions. Node-edge network approaches illustrate which nodes form a sub-network, which nodes interact within these sub-networks, and the types of interactions between each node, giving an all encompassing view of the sub-network. Matrix-based approaches provide a broader view of interactions, offering a tool for visualizing not only how similar nodes and clusters are to each other, but also the interactions nodes share outside of their individual clusters.

## Conclusion

Current network analyses of disease are still highly focused on gene and protein-based networks, neglecting environmental and drug effects that contribute to the pathophysiology of a disease or sets of diseases. Our proposed methods integrate both the chemical and disease entities into network and matrix-based analyses, allowing for a more complete systems understanding of the underlying biology. With this addition of multiple different entity types comes the lack of a gold standard for identifying specific genes, chemicals, and diseases that should cluster together, providing a similar role as the curated regulatory and pathway networks used to establish accuracy in protein-protein and gene-gene network analyses.

In order to better investigate complex and sparse networks, such as the respiratory disease interactome, a multi-method approach utilizing methods proven effective in gene-gene and protein-protein network-based analyses has proven useful to elucidate and investigate different network properties and the underlying biological context. In this case we have used two approaches: a node-edge-based clustering coefficient with Jaccard similarity comparison approach applied to traditional networks, and a matrix-based Pearson’s correlation coefficient with hierarchical clustering approach. This allows identification of closely interacting diseases, chemicals, and genes, as well as similar interaction profiles either within or between these same elements of interest. These two approaches help facilitate investigations on the underlying biology for a given disease, pathophysiology similarities across diseases, and chemicals that may have a therapeutic indication outside of their original use.

The shared interactome of four therapeutic antibiotics (ofloxacin, amoxicillin clavulanate, clarithromycin, and azizthromycin, (Figure 9) allows for an inference of interaction between azithromycin and tuberculosis based upon the interaction profile of a cluster generated by hierarchically clustering a Pearson’s correlation coefficient matrix. This profile represents the layering of diseases, chemicals, genes and the interactions between them, showing that while azithromycin has no known anti-Mycobacterium tuberculosis properties, it does have pro-host immune properties that may have therapeutic merit for tuberculosis treatment.

These methods are also useful for finding drug targets. The shared interactome of pulmonary fibrosis and asthma (Figure 1) demonstrates that Th2 cell inflammation is an important factor in both of these diseases, where a drug that improves the outcomes in one of these diseases may also be useful for the other disease. Looking at these interactomes provides a broader context for drug discovery and drug repurposing.

Chemical, gene, and disease interactomes offer a novel approach to not only identify shared biology among diseases, but also offer a method for identifying possible new drug targets and repurposed drug strategies. Layering additional interaction information, additional databases, and additional analysis techniques will allow for a more complete systems-based analysis that will extend to any complex disease interactome.

## Methods

### Network generation

Respiratory diseases and the curated chemical and genes interactions with these diseases were extracted from CTD using the January 9, 2012 database version [4]. Curated chemical-gene interactions were extracted from batch queries using the chemicals and genes associated with respiratory diseases. Genes, chemicals, and their associated links that did not contain a link to a respiratory disease were removed from the list. Duplicates of gene-chemical and chemical-gene links were also removed from this list. Gene-gene interactions were established using the April 13, 2010 version of the HPRD database [1]. Genes and their associated interactions were excluded from the list if they did not contain a direct link to a respiratory disease. These interactions were further specialized by including only chemicals with therapeutic interactions to respiratory diseases in a therapeutic network, with the “therapeutic” name stemming from including only chemicals with at least one therapeutic indication. The therapeutic indication for a chemical is determined from the by the direct evidence field from CTD. Genes were then excluded if they did not contain a link to one of these therapeutic chemicals. Chemical-chemical links and chemical-gene interaction characteristics for the therapeutic network were established using the February 10, 2012 version of CTD [4]. Chemical-chemical links were established using co-occurrence of chemicals in chemical-gene interactions. A chemical was established as co-occurring when a secondary chemical appeared in the interaction characteristics of chemical-gene interactions. A triple was stored for each interaction, including both interacting nodes and the type of interaction between them.

### Network and matrix visualization

A file containing the triples of interactions and a file containing the type of node (chemical, gene, disease) were loaded into Cytoscape [22]. Nodes were colored based upon their type, with chemicals represented as blue, genes as black, and diseases and orange. Interactions were colored based upon interaction characteristics, with positive interactions as green, negative interactions as red, mixed interactions as purple, and additional characteristics as increasing intensity.

A binary interaction matrix between nodes was created using the network construction file containing interaction triples. A value of 1 was used for any interaction type between nodes and a value of 0 was used for a lack of interaction between nodes. This binary interaction matrix was visualized by creating a bitmap of clustered interactions and the resulting dendrograms by using TreeView [34].

### Network and matrix clusters

MCODE, a Cytoscape plugin, was used to generate each of the sub-networks [17,22]. A degree cutoff of 2, and node score cutoff of 0.2, a k-core of 2, and a max depth of 100 were used as the MCODE parameters for generating clusters.

Cluster 3.0 was used to generate clusters for this matrix [33]. An uncentered similarity with average linkage was used to calculate the hierarchical clustering. Similarity scores of 0.4 and 0.7 were used for creating clusters, based upon inflection points Figure 7.

### Jaccard similarity

Jaccard similarity coefficients were generated for both the therapeutic network and for sub-networks using the following formula: $\frac{\text{Node}1\;\cap \;\text{Node}2}{\text{Node}1+\text{Node}2-\left(\text{Node}1\;\cap \;\text{Node}2\right)}$. This formula calculates the intersection of the two sets divided by their union. A set, in all cases, is all the nodes that interact with a given node, including any self-interactions. The intersection of two nodes is all shared interactions between the two nodes, with the union of the two nodes being all the nodes that interact with at least one of the nodes of interest. For the entire therapeutic network, a Mann–Whitney U test was run with the alternative hypothesis that linked nodes are more similar than unlinked nodes. For sub-networks, ranks of Jaccard coefficients were calculated using the individual sub-network that a node pair come from and then compared to the evidence of there being an interaction.

### Network stability

Sub-networks were used to assess the stability of the network in respect to changes in Jaccard coefficient. For a given sub-network, an additional network was generated for each missing edge. In each of these networks one additional edge was added between two existing unlinked nodes, creating a unique set of networks. Jaccard coefficients were then generated for each additional network. Two-sample Kolmogorov-Smirnov tests were used to assess whether or not the distribution of the original sub-network and the altered sub-networks was shifted. This was done for each of the sub-networks and their corresponding altered networks. The null hypothesis was that the Jaccard coefficient distribution of the network with an additional edge is the same as the unaltered sub-network, with the alternative hypothesis being that the distribution is shifted.

### Programming

Original network parsing to establish interactions between nodes was done using perl version 5.12.4 on Mac OSX 10.7. This includes parsing interactions between genes, chemicals, and diseases, finding which chemicals have co-interactions with genes, finding unique interactions and directional interactions between chemicals and genes, finding interaction characteristics for disease-gene and disease-chemical interactions, and selecting inclusion criteria for interactions of interest to develop each network.

Further network parsing, matrix construction, and dendrogram parsing was done using C#/.NET 4.0 on a Windows 7 machine. This includes finding specific interaction characteristics for chemical-gene and chemical-chemical interactions, construction of the interaction matrix, visualization of the interaction matrix, and extracting clusters based upon a threshold from the output from Cluster 3.0.

## Competing interests

The authors declare that they have no competing interests.

## Authors’ contribution

BG – Co-conceived the Project, Methods development, coding, paper writing. GD – Methods development, coding, paper editing. GC – Methods development. MS – Conceived the Project, Supervised the Project, Methods development, paper editing. All authors read and approved the final manuscript.

## Supplementary Material

Additional file 1: Table S1 Whole Respiratory Network. Click here for file

Additional file 2: Table S2 Therapeutic Network. Click here for file

Additional file 3: Table S3 MCODE Clusters. Click here for file

Additional file 4: Table S4 Hierarchical Clusters. Click here for file

## References

[B1] Keshava Prasad TS Goel R Kandasamy K Keerthikumar S Kumar S Mathivanan S Telikicherla D Raju R Shafreen B Venugopal A Balakrishnan L Marimuthu A Banerjee S Somanathan DS Sebastian A Rani S Ray S Harrys Kishore CJ Kanth S Ahmed M Kashyap MK Mohmood R Ramachandra YL Krishna V Rahiman BA Mohan S Ranganathan P Ramabadran S Chaerkady R Pandey A Human protein reference database–2009 update Nucleic Acids Res 2009 37 D767 D772 10.1093/nar/gkn892 18988627 PMC2686490

[B2] Baxevanis AD Searching Online Mendelian Inheritance in Man (OMIM) for information for genetic loci involved in human disease Current protocols in bioinformatics 2003 35 9.13 1-9 13.15 10.1002/0471250953.bi0102s27 19728286

[B3] Goto S Okuno Y Hattori M Nishioka T Kanehisa M LIGAND: database of chemical compounds and reactions in biological pathways Nucleic Acids Res 2002 30 402 404 10.1093/nar/30.1.402 11752349 PMC99090

[B4] Davis AP King BL Mockus S Murphy CG Saraceni-Richards C Rosenstein M Wiegers T Mattingly CJ The comparative toxicogenomics database: update 2011 Nucleic Acids Res 2011 39 D1067 D1072 10.1093/nar/gkq813 20864448 PMC3013756

[B5] Lamb J Crawford ED Peck D Modell JW Blat IC Wrobel MJ Lerner J Brunet JP Subramanian A Ross KN Reich M Hieronymus H Wei G Armstrong SA Haggarty SJ Clemons PA Wei R Carr SA Lander ES Golub TR The connectivity Map: using gene-expression signatures to connect small molecules, genes, and disease Science 2006 313 1929 1935 10.1126/science.1132939 17008526

[B6] Tong AH Drees B Nardelli G Bader GD Brannetti B Castagnoli L Evangelista M Ferracuti S Nelson B Paoluzi S Quondam M Zucconi A Hogue CW Fields S Boone C Cesareni G A combined experimental and computational strategy to define protein interaction networks for peptide recognition modules Science 2002 295 321 324 10.1126/science.1064987 11743162

[B7] American Lung Association State of lung disease in diverse communities 2010

[B8] Kaneko Y Yatagai Y Yamada H Iijima H Masuko H Sakamoto T Hizawa N The search for common pathways underlying asthma and COPD Int J Chron Obstruct Pulmon Dis 2013 8 65 78 23378757 10.2147/COPD.S39617 PMC3558318

[B9] Dockstader K Nunley K Karimpour-Fard A Medway A Nelson P Port JD Liggett SB Bristow MR Sucharov CC Temporal analysis of mRNA and miRNA expression in transgenic mice overexpressing Arg- and Gly389 polymorphic variants of the beta1-adrenergic receptor Physiol Genomics 2011 43 1294 1306 10.1152/physiolgenomics.00067.2011 21954455 PMC3233820

[B10] Janjic V Przulj N Biological function through network topology: a survey of the human diseasome Brief Funct Genomics 2012 11 522 532 10.1093/bfgp/els037 22962330 PMC7109924

[B11] Islam MF Hoque MM Banik RS Roy S Sumi SS Hassan FM Tomal MT Ullah A Rahman KM Comparative analysis of differential network modularity in tissue specific normal and cancer protein interaction networks J Clin Bioinformatics 2013 3 19 10.1186/2043-9113-3-19 PMC3852839 24093757

[B12] Barzel B Barabasi AL Network link prediction by global silencing of indirect correlations Nat Biotechnol 2013 31 720 725 10.1038/nbt.2601 23851447 PMC3740009

[B13] Iorio F Saez-Rodriguez J Bernardo D Network based elucidation of drug response: from modulators to targets BMC Syst Biol 2013 7 139 10.1186/1752-0509-7-139 24330611 PMC3878740

[B14] Wang X Wei X Thijssen B Das J Lipkin SM Yu H Three-dimensional reconstruction of protein networks provides insight into human genetic disease Nat Biotechnol 2012 30 159 164 10.1038/nbt.2106 22252508 PMC3708476

[B15] Yeh SH Yeh HY Soo VW A network flow approach to predict drug targets from microarray data, disease genes and interactome network - case study on prostate cancer J Clin Bioinformatics 2012 2 1 10.1186/2043-9113-2-1 PMC3285036 22239822

[B16] Gulbahce N Yan H Dricot A Padi M Byrdsong D Franchi R Lee DS Rozenblatt-Rosen O Mar JC Calderwood MA Baldwin A Zhao B Santhanam B Braun P Simonis N Huh KW Hellner K Grace M Chen A Rubio R Marto JA Christakis NA Kieff E Roth FP Roecklein-Canfield J Decaprio JA Cusick ME Quackenbush J Hill DE Münger K Vidal M Barabási AL Viral perturbations of host networks reflect disease etiology PLoS Comput Biol 2012 8 e1002531 10.1371/journal.pcbi.1002531 22761553 PMC3386155

[B17] Bader GD Hogue CW An automated method for finding molecular complexes in large protein interaction networks BMC Bioinforma 2003 4 2 10.1186/1471-2105-4-2 PMC149346 12525261

[B18] Enright AJ Van Dongen S Ouzounis CA An efficient algorithm for large-scale detection of protein families Nucleic Acids Res 2002 30 1575 1584 10.1093/nar/30.7.1575 11917018 PMC101833

[B19] Nguyen P Srihari S Leong H Identifying conserved protein complexes between species by constructing interolog networks BMC Bioinforma 2013 14 S8 10.1186/1471-2105-14-S16-S8 PMC4098725 24564762

[B20] Maetschke SR Madhamshettiwar PB Davis MJ Ragan MA Supervised, semi-supervised and unsupervised inference of gene regulatory networks Brief Bioinform 2013 15 195 211 23698722 10.1093/bib/bbt034 PMC3956069

[B21] Trinidad JC Thalhammer A Burlingame AL Schoepfer R Activity-dependent protein dynamics define interconnected cores of co-regulated postsynaptic proteins Mol Cell Proteomics: MCP 2013 12 29 41 10.1074/mcp.M112.019976 PMC3536907 23035237

[B22] Smoot ME Ono K Ruscheinski J Wang PL Ideker T Cytoscape 2.8: new features for data integration and network visualization Bioinformatics 2011 27 431 432 10.1093/bioinformatics/btq675 21149340 PMC3031041

[B23] Lee CG Cho SJ Kang MJ Chapoval SP Lee PJ Noble PW Yehualaeshet T Lu B Flavell RA Milbrandt J Homer RJ Elias JA Early growth response gene 1-mediated apoptosis is essential for transforming growth factor beta1-induced pulmonary fibrosis J Exp Med 2004 200 377 389 10.1084/jem.20040104 15289506 PMC2211975

[B24] Wills-Karp M Interleukin-13 in asthma pathogenesis Immunol Rev 2004 202 175 190 10.1111/j.0105-2896.2004.00215.x 15546393

[B25] Madhamshettiwar PB Maetschke SR Davis MJ Reverter A Ragan MA Gene regulatory network inference: evaluation and application to ovarian cancer allows the prioritization of drug targets Genome Med 2012 4 41 10.1186/gm340 22548828 PMC3506907

[B26] Becker JC Muller-Tidow C Stolte M Fujimori T Tidow N Ilea AM Brandts C Tickenbrock L Serve H Berdel WE Domschke W Pohle T Acetylsalicylic acid enhances antiproliferative effects of the EGFR inhibitor gefitinib in the absence of activating mutations in gastric cancer Int J Oncol 2006 29 615 623 16865277 10.3892/ijo.29.3.615

[B27] Selvendiran K Bratasz A Tong L Ignarro LJ Kuppusamy P NCX-4016, a nitro-derivative of aspirin, inhibits EGFR and STAT3 signaling and modulates Bcl-2 proteins in cisplatin-resistant human ovarian cancer cells and xenografts Cell Cycle 2008 7 81 88 10.4161/cc.7.1.5103 18196976 PMC2890223

[B28] Van Dyke AL Cote ML Prysak G Claeys GB Wenzlaff AS Schwartz AG Regular adult aspirin use decreases the risk of non-small cell lung cancer among women Canc Epidemiol Biomarkers Prev: a Pub of the Am Assoc for Cancer Res, cosponsored by the Am Soc of Preventive Oncolgy 2008 17 148 157 10.1158/1055-9965.EPI-07-0517 PMC3771076 18187393

[B29] Braun DP Bonomi PD Taylor SG Harris JE Modification of the effects of cytotoxic chemotherapy on the immune responses of cancer patients with a nonsteroidal, antiinflammatory drug, piroxicam. A pilot study of the Eastern Cooperative Oncology Group J Biol Response Modif 1987 6 331 345 3037033

[B30] Palmerini E Fan K Yang K Risio M Edelmann W Lipkin M Biasco G Piroxicam increases colon tumorigenesis and promotes apoptosis in Mlh1 +/− /Apc1638(N/+) mice Anticancer Res 2007 27 3807 3812 18225536

[B31] Vizza CD Rocca GD Roma AD Iacoboni C Pierconti F Venuta F Rendina E Schmid G Pietropaoli P Fedele F Acute hemodynamic effects of inhaled nitric oxide, dobutamine and a combination of the two in patients with mild to moderate secondary pulmonary hypertension Critical Care 2001 5 355 361 10.1186/cc1069 11737925 PMC96124

[B32] Zaman N Li L Jaramillo ML Sun Z Tibiche C Banville M Collins C Trifiro M Paliouras M Nantel A O'Connor-McCourt M Wang E Signaling network assessment of mutations and copy number variations predict breast cancer subtype-specific drug targets Cell Rep 2013 5 216 223 10.1016/j.celrep.2013.08.028 24075989

[B33] de Hoon MJ Imoto S Nolan J Miyano S Open source clustering software Bioinformatics 2004 20 1453 1454 10.1093/bioinformatics/bth078 14871861

[B34] Saldanha AJ Java Treeview--extensible visualization of microarray data Bioinformatics 2004 20 3246 3248 10.1093/bioinformatics/bth349 15180930

[B35] Metheny-Barlow LJ Flynn B van Gijssel HE Marrogi A Gerwin BI Paradoxical effects of platelet-derived growth factor-A overexpression in malignant mesothelioma. Antiproliferative effects in vitro and tumorigenic stimulation in vivo Am J Respir Cell Mol Biol 2001 24 694 702 10.1165/ajrcmb.24.6.4334 11415934

[B36] Mandhane SN Shah JH Bahekar PC Mehetre SV Pawar CA Bagad AS Chidrewar GU Rao CT Rajamannar T Characterization of anti-inflammatory properties and evidence for no sedation liability for the novel antihistamine SUN-1334H Int Arch Allergy Immunol 2010 151 56 69 10.1159/000232571 19672097

[B37] Truong-Tran AQ Ruffin RE Foster PS Koskinen AM Coyle P Philcox JC Rofe AM Zalewski PD Altered zinc homeostasis and caspase-3 activity in murine allergic airway inflammation Am J Respir Cell Mol Biol 2002 27 286 296 10.1165/rcmb.2001-0014OC 12204890

[B38] Chambers HF Kocagoz T Sipit T Turner J Hopewell PC Activity of amoxicillin/clavulanate in patients with tuberculosis Clin Infect Dis: an official publication of the Infectious Dis Soc of America 1998 26 874 877 10.1086/513945 9564467

[B39] Nadler JP Berger J Nord JA Cofsky R Saxena M Amoxicillin-clavulanic acid for treating drug-resistant Mycobacterium tuberculosis Chest 1991 99 1025 1026 10.1378/chest.99.4.1025 1901260

[B40] Agarwal S To assess the clinical efficacy of azithromycin and capreomycin in the threatment of multi-drug resistant pulmonary tuberculosis Chest 2004 126 752S

[B41] Watt B Rayner A Harris G Comparative activity of azithromycin against clinical isolates of mycobacteria J Antimicrob Chemother 1996 38 539 542 10.1093/jac/38.3.539 8889727

